# Are frequent measurements in back pain research harmful? Two comparisons of back pain in groups with or without frequent follow-up

**DOI:** 10.1186/s12998-018-0220-0

**Published:** 2018-12-11

**Authors:** Lise Hestbaek, Annette Christina Saxtorph, Carl-Emil Krogsgaard-Jensen, Alice Kongsted

**Affiliations:** 1 Nordic Institute of Chiropractic and Clinical Biomechanics, Odense, Denmark; 2 Dept. of Sports Science and Clinical Biomechanics, University of Southern Denmark, Odense, Denmark; 3 Private Chiropractic Practice, Copenhagen, Denmark

**Keywords:** Monitoring, Back pain, Neck pain, Frequent measures, Pain intensity

## Abstract

**Background:**

Frequent measures are becoming increasingly used to evaluate the course of spinal pain. However, it is not known whether this type of continuous follow-up in itself has implications for people’s experience of pain. Therefore this article examines a potential impact of frequent follow-up using SMS reporting on the report of pain, based on results from two previous studies of spinal pain.

**Methods:**

We examined two sets of cohorts, where each set was comparable in all other aspects, but one cohort in each set had been followed with weekly SMS-questions about the presence of spinal pain for 6 years and 1 year, respectively, whereas the other cohort had not answered any questions for research purposes before. At the end of the follow-up period, two cohorts, consisting of pupils from 5th and 6th grade, completed the Young Spine Questionnaire about spinal pain, one cohort in 2010 and the other in 2014. The other set of cohorts, consisting of low back pain patients in primary care, completed an extensive questionnaire about their back pain (2011 to 2013).

**Results:**

In both sets of cohorts there was a statistically significant difference in pain intensity with the pupils/patients who had been subject to frequent follow-up over long periods of time reporting lower intensity of pain. Other differences were small and not statistically significant.

**Conclusion:**

Since the data were not optimally suited for the purpose of these analyses, the results should obviously be interpreted with caution, but they do not support a theory about increased attention leading to increased awareness, which in turn will lead to increased pain. On the contrary, participants reported lower levels of pain when belonging to the samples that had been subject to frequent follow-up by SMS-track over long periods of time.

## Introduction

Advances in technology have given rise to numerous possibilities for monitoring anywhere in society. In medical research, communication technologies have opened a feasible way to close monitoring of health conditions, either continuously as in blood pressure surveillance [1], or frequently as in diabetes treatment [2].

Back and neck pain is characterized by remissions and exacerbations [3–6] and therefore it is difficult to gain an in-depth understanding of its course through traditional research methods such as questionnaires or interviews at a few fixed time-points. Hence, frequent measures are becoming increasingly used to evaluate the course of back and neck pain, most often using brief daily or weekly automated text messages (SMS-track) [6, 7]. SMS-track is an efficient way to obtain frequent data and has been proven valid [8] when compared to telephone interviews and has resulted in response rates above 90% in some settings [9]. However, it is not known whether this type of continuous follow-up in itself has implications for the patients’ experience of pain.

It is possible that a continuous reminder of a problem will make the person adhere to a self-perception as a patient, thus rendering recovery less likely. On the other hand, an increased registration will help to quantify the problem, which may lead to decreased concern by illustrating the extent, and thereby also the limitation or even improvement, of the problem. As with all types of outcomes, it is important to realize if and to what extent, the registration itself alters the outcome. We are not aware of well-designed studies which can illustrate this with regard to frequent measures of back and neck pain.

We have gathered data with SMS-track in both a population-based cohort of schoolchildren and in a clinical sample of low back pain patients. Through comparison with study subjects who completed baseline and follow-up questionnaires but did not receive text messages, these data can be used to give a preliminary indication of whether the increased attention through frequent follow-up will lead to increased attention and thus increased reporting of back pain.

## Methods

### Cohort 1a (schoolchildren without SMS-track)

These data originates from the SPACE study. SPACE was a school-based cluster-randomized controlled trial involving 14 schools in the Region of Southern Denmark. The main aim of SPACE was to investigate how physical environment combined with organizational initiatives could promote physical activity in adolescence. All 5th and 6th grade students (11–13 y.o.a.) at the 14 schools were invited to participate. There were no exclusion criteria. For a comprehensive description, see the SPACE protocol [10]. At baseline (April to June 2010), the participants completed an electronic questionnaire (e-survey), including the Young Spine Questionnaire (YSQ) [11], during school time, observed by a teacher who also ensured that there were no interactions between participants.

### Cohort 1b (schoolchildren with SMS-track)

This cohort is nested in a six-year prospective longitudinal study of schoolchildren, the CHAMPS Study-DK. This was a cluster-randomized controlled trial involving all schools in one municipality in the Region of Southern Denmark. The main aim of the CHAMPS study was to investigate the effect of more physical education in the curriculum. Children from 0th to 4th grade from 13 primary schools were invited to participate. The CHAMPS Study-DK commenced in August 2008 and the data collection ended July 2014 (end of term for 5th to 9th grade). Children could enter or leave the study at any time during the study period, and thus be enrolled for up to 6 years. One of the parents of participating children received weekly text messages inquiring about the child’s musculoskeletal complaints. The protocol for CHAMPS Study-DK has been published elsewhere [12].

Towards the end of the study (June 2014), a subset of the included children completed the YSQ. A total of 500 questionnaires were divided between the schools and distributed by the teachers. As in the SPACE study, the children filled in the questionnaires during school hours, observed by a teacher, who also collected the questionnaires. As the summer holiday was approaching, the schools were busy and therefore it was left to the discretion of the schools to choose which classes to involve, based on convenience.

To compare with the sample in cohort 1a, only results from children attending 5th or 6th grade at the time of questioning are used in this report.

### Outcome for comparison of the two school-based cohorts

The YSQ includes identical questions for the three spinal regions separately. Using the neck questions as example, the first question was: “Have you ever had pain in your neck?” (“often”/ “sometimes”/ “once or twice”/ “never”). This was followed by a question about pain intensity (Faces Pain Scale- revised [13]) for the worst pain experienced, which was converted into a 0–10 point scale. The questions were repeated for the mid back and low back. In both cohorts, the questionnaire was given to the children during school hours, thus the major difference between the two cohorts is that the one (1a) had never been asked about spinal problems for research purposes before, whereas the other (1b) had received weekly text messages for up to 6 years prior to answering the YSQ.

Prevalence of neck pain (NP), mid back pain (MBP), low back pain (LBP) or any type of spinal pain (SP), defined as ‘often’ or ‘sometimes’, was used as the outcome in the comparative analyses as well as mean pain intensity for the region of the spine with the highest pain intensity.

### Cohort 2 (LBP patients with and without SMS-track)

This cohort reports on data from a prospective cohort study aimed at identifying course patterns, subgroups and prognostic factors in patients with low back pain (LBP)*.* The study is described in detail elsewhere [14]. The original study included LBP patients from both general practice and chiropractic practice in Denmark to reflect patients in primary healthcare from 2010 to 2014, but this report will only analyze data from general practitioners (GP), since no data are available from chiropractic patients, who did not consent to frequent follow-up. Inclusion criteria were LBP with or without radiating pain, age 18–65 years and access to a mobile phone. Exclusion criteria were pregnancy, suspicion of serious pathology and inability to read and write Danish. In connection with a consultation for LBP, the GP filled out a brief baseline form and the patients received an envelope containing written information about the prospective study and a baseline questionnaire, including written consent. All patients who agreed to participate in close monitoring for a year were enrolled in a weekly follow-up, using SMS-track. The SMS questions were: “How many days did you have low back pain during the last week?” and “How intense was the pain typically on a scale from 0 to 10?” The second question was only asked if the patient answered one or more days in response to the first question. Furthermore, the patients received a follow-up questionnaire 12 months after the initial LBP consultation. Both SMS-track and questionnaires were administered by the research team without involvement of the practitioners. These patients will be referred to as cohort 2b.

It was decided also to send the 12 months follow-up questionnaire to the patients that participated in the baseline registration from the GPs, but had *not* participated in the SMS track. For these patients, data recorded by the GP at the baseline visit was available, but there had been no contact between the patient and the research team for the 12 months following the initial visit. These patients will be referred to as cohort 2a.

### Outcome for comparison of the LBP-patients

‘Bothersomeness of LBP during the last two weeks’ (5-point Likert scale), ‘more than 30 days of LBP during the past year’, Roland Morris proportional score (RMDQ) [15] and pain intensity the past week (0–10 box scale), all from the 12 months follow-up questionnaire, was used as outcomes in the comparative analyses.

### Analyses

Baseline variables were compared between the cohorts where possible. For cohort 1a and 1b, variables available in both cohorts were school grade (as proxy for age) and sex; for cohorts 2a and 2b, they were age, sex, pain below the knee, duration of initial LBP episode and number of previous LBP episodes. Next, outcomes were reported with 95% confidence intervals (CI) for comparison between samples. Finally, to account for potential confounders, multiple regression models were constructed to estimate the effect of being closely monitored by frequent SMS-questions. Pain intensity and RMDQ, as continuous variables, were tested in linear regressions. For consistency and ease of interpretation, they were also dichotomized, as was ‘Bothersomeness of LBP during the last two weeks’, as described in Table 2 and included in logistic regressions. Baseline variables were included as confounders if there were differences between the cohorts.

**Fig. 1:**
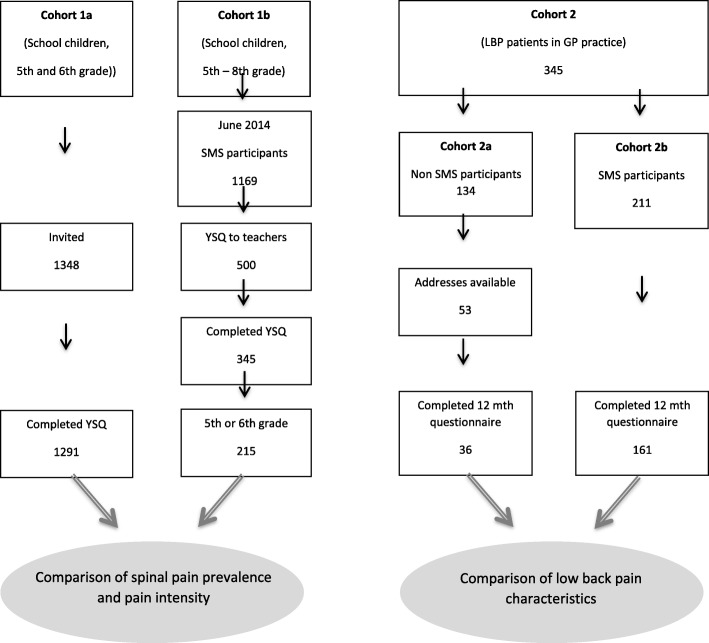
Flowchart

An overview of the study is provided in Figure 1.

## Results

### Comparison of the two school-based cohorts

In sample 1a, who had never been involved in research prior to receipt of the questionnaire, 1291 students from 5th and 6th grade returned completed questionnaires. In sample 1b, who had received weekly text messages for up to 6 years, 345 children returned completed questionnaires; 215 of these attended 5th or 6th grade and was therefore comparable to sample 1a.

The children in sample 1a were slightly younger and more likely to be boys than the children in sample 1b. Thus, both grade and gender were included in the regression models.

Results of the comparisons are shown in Table 1. The mean pain intensity was lower for the children in sample 1b, who had been followed with SMS-track, than in sample 1a, but none of the other outcomes showed any differences. This was also reflected in a statistically significant odds ratio (OR) for reporting a low pain score in sample 1b in reference to sample 1a, but no difference in odds for reporting prevalence of spinal pain (Table 2). Lower pain intensity with SMS tracking than without was also significant in the linear regression model when adjusted for grade and sex (β = − 0.88 (95%CI: -1.35;-0.40), *p* < 0.000).

**Table 1:** Age, sex and spinal pain characteristics for the four cohorts. Where nothing else is noted, results are reported as proportions with 95% confidence intervals (CI)

	Cohort 1 (no SMS) *n* = 1291	Cohort 1b (SMS) *n* = 215		Cohort 2a (no SMS) *n* = 36	Cohort 2b (SMS) *n* = 161
5th grade 6th grade	50% 50%	40% 60%	Age at baseline mean (95%CI)	48 (45–52)	47 (45–48)
Male	52% (49–54%)	43% (36–50%)	Male	47% (30–65%)	41% (33–49%)
NP^a^	36% (33-39%)	36% (29–42%)			
MBP^a^	24% (21-26%)	28% (22–34%)	Bothersomeness past 2 weeks^c^ Median (IQR)	3 (2–4)	2 (2–3)
LBP^a^	16% (14-18%)	24% (18–30%)	> 30 days of LBP previous year	72% (54–86%)	62% (54–70%)
SP	46% (44–49%)	50% (43–56%)	RMDQ^d^ mean (95% CI)	39 (29–49)	29 (24–33)
Pain intensity^b^ mean (95% CI)	4.74 (4.46–5.02)	3.89 (3.52–4.27)	VAS past week mean (95% CI)	4.61 (3.43–5.79)	3.23 (2.81–3.65)

^a^‘often or some times’

^b^FPS-r converted to a 0 to 10 scale for the spinal region with the highest reported pain intensity (only for those with a report of pain)

^c^five-point Likert scale

^d^Proportional score on the Roland Morris Disability Questionnaire

**Table 2:** Odd ratios (OR) for having a negative outcome if followed by weekly text messages in contrast to no follow-up

	Schoolchildren OR (95% CI) for cohort 1b^a^	LBP patients OR (95% CI) for cohort 2b^b^
SP ^‘^often’ or ‘some times’	1.13 (0.85–1.52)	-
Pain intensity above 3/10	**0.64 (0.45–0.91)**	**0.39 (0.19–0.83)**
Bothersomeness past 2 weeks^c^	-	0.55 (0.27–1.16)
> 30 days of LBP previous year	-	0.63 (0.29–1.41)
RMDQ^d^	-	0.71 (0.31–1.61)

^a^cohort 1a as reference, adjusted for grade and sex

^b^cohort 2a as reference, no adjustments made

^c^3–5 on a five-point Likert scale

^d^> 60 on the proportional score on the Roland Morris Disability Questionnaire

Statistically significant findings indicated with boldface

### Comparison of the two LBP-patient cohorts

There were no differences between the two cohorts in relation to baseline variables (age, sex, pain below the knee, duration of episode and previous episodes) (data not shown) and therefore no confounders were added to the regression model.

For all four outcomes, cohort 2b, which had been followed for a year with SMS-track, tended to have better outcomes than cohort 2a, who had not. However, none of the differences were statistically significant (Table 1).

Similarly to the child-cohorts, the logistic regression models showed a statistically significant OR for reporting a low level of pain in the cohort with close monitoring compared to the cohort without monitoring, whereas there was no statistically significant effect of SMS-track on the other outcomes (Table 2). For the continuous outcomes, linear regression also demonstrated significantly lower pain intensity in cohort 2b as compared to 2a (β = − 1.38 (95%CI: -2.42;-0.34), *p* = 0.010) but not a significant difference in disability (β = − 10.29 (95%CI: -21.23;0.65), *p* = 0.065).

## Discussion

Our results do not support a theory about increased attention leading to increased awareness, which in turn will lead to increased pain. On the contrary, participants reported lower levels of pain when belonging to the samples that had been subject to frequent follow-up by SMS-track over long periods of time.

Since the data were not optimally suited for the purpose of these analyses, the results should obviously be interpreted with caution and studies designed for the specific purpose should be conducted. However, similar findings in two different patient populations do add to the robustness of the findings.

### Limitations

- Participation: There were no differences between the baseline variables measured by the GP in cohorts 2a and 2b, but there are likely to be unmeasured differences, leading to participation in the follow-up study. These differences could be potential risk factors for poor prognosis and thus explain all or part of the differences reported 12 months later.
- Sample size: Cohort 2a is very small and since the results are consistently in favor of the SMS-group, these differences might be significant in a larger sample.
- Parental SMS reporting: In cohort 1b, the parents answered the SMS, whereas the children themselves answered the YSQ. It was assumed that the parents would ask the children about the pain before answering the SMS, but it is unknown whether this actually happened. If not, the SMS-track does not have the potential to influence the children’s self-report of pain.

## Conclusion

Since the data were not optimally suited for the purpose of these analyses, the results should obviously be interpreted with caution, but they do not support a theory about increased attention leading to increased awareness, which in turn will lead to increased pain. Therefore studies better designed to answer this question should be conducted, but until better evidence becomes available, there does not seem to be reason to avoid frequent follow-up using SMS-reporting in musculoskeletal research from fear of creating or maintaining pain through attention.

## Abbreviations

CHAMPS: The Childhood Health, Activity and Motor Performance School Study
CI: Confidence interval
GP: General practitioner
LBP: Low back pain
MBP: Mid back pain
NP: Neck pain
RMDQ: Roland Morris Disability Questionnaire
SMS: Short Message Services
SP: Spinal pain
SPACE: School site, Play spot, Active transport, Club fitness and Environment
YSQ: Young spine questionnaire
